# Multi‐factor analysis of the quality of cellar mud of Luzhou‐flavor liquor in Yibin production area

**DOI:** 10.1002/fsn3.4174

**Published:** 2024-05-30

**Authors:** Baolin Han, Weitao Zhou, Rangfang Chen, Shulin Tian, Hucheng Gong, Yu Wang, Qiang Xu, Minghong Bian

**Affiliations:** ^1^ Sichuan University of Science & Engineering Yibin China

**Keywords:** microbial community analysis, mud pit of Luzhou‐flavor baijiu, physicochemical property, volatile substances

## Abstract

The aim of this study was to conduct a thorough scientific investigation into the similarities and differences in the quality of the cellars of different Luzhou‐flavor liquor wineries in Yibin production area and the reasons for them. This study analyzed cellar mud samples from five wineries in Yibin production area. The analysis of volatile flavor compounds was carried out using headspace solid‐phase microextraction and gas chromatography–mass spectrometry. The bacterial and archaeal community structures and their correlations were analyzed by high‐throughput sequencing. The study indicates that the Distillery A had the highest levels of ammonium nitrogen and effective phosphorus, Distillery F had the highest humus levels, and Distillery I had the highest pH levels. The community structure of the principal bacterial and archaeal communities in the five subterranean clays exhibited similarity, and all samples were dominated by Firmicutes as the primary bacterial group. However, there was variation in bacterial abundance. The cellar mud also has obvious regional differences, and there are three genera of differentially dominant archaea in the archaea. In summary, significant differences were observed in the physicochemical indexes of bacterial and archaeal abundance across all five samples. These differences led to variations in both the content and composition of volatile constituents.

## Graphical abstract

The data studied in this paper show that the interaction of factors, such as climatic conditions, soil type, vegetation cover, topography, and agricultural activities, has a complex dynamic and interactive effect on the physicochemical indexes, microbial population structure, and content of flavor substances in the cellar soil. The cellar soil can be adjusted and optimized according to the data and differences in subsequent research and actual production.

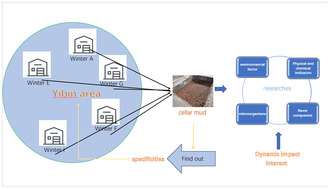

## 1 INTRODUCTION

Luzhou‐flavored liquor is defined as liquor with a distinct aroma of ethyl caproate made from raw materials, such as sorghum, wheat, glutinous rice, and other grains, via solid‐state fermentation, distillation, aging, and blending (Zhang, 2019). Luzhou‐flavored liquor is known for its “rich cellar aroma, sweetness and mellowness, harmonious fragrance and, long aftertaste.” It has become one of the most popular liquors in China due to its unique flavor and taste (Liu & Sun, 2018; Liu et al., 2013; Yuchen, 2022). Yibin City, Sichuan Province, China, is the first city on the Miles Yangtze River; it is located in the Golden Triangle region of China's liquor. It serves as a significant area for the production of China's strongly flavored liquor, with the liquor industry being its traditional and primary source of advantage and strength. Cellar mud is an important factor in the brewing process of concentrated white wine. Cellar mud quality is directly related to the brewing of white wine quality and taste; the study of cellar mud has been one of the hot spots in the field of white wine brewing (Luo et al., 2014).

The cellar mud of concentrated white wine is one of the main sources of microorganisms for white wine brewing (Du et al., 2013), and it is a special soil sample whose quality has an important impact on the quality and taste of white wine (Qun, 2020). Cellar mud contains a variety of nitrogen‐containing, sulfur‐containing organic substances, minerals, etc. The content, type, and composition of these substances can affect the quality of the cellar mud and the cellar microbial growth and metabolism, especially for the quality of white wine, has an important impact on the microbial group (Liu et al., 2018). Functional microbial species and quantity in the mud found in the cellar are the primary determinants of the mud quality (Hu et al., 2021; Liao et al., 2010). In actual production, these microorganisms decompose raw materials in starch protein and accelerate the microbial metabolism of acid‐alcohol substances into esters while producing flavor substances, giving white wine a unique flavor.

The physical and chemical indexes of the cellar mud and the production of flavor compounds are closely linked to the microflora composition; *Lactobacillus* was the predominant genus found in the old cellar mud, whereas the dominant genus in the new cellar mud was bacteria that produced hexanoic acid, and the most significant environmental factors were lactate, pH, and soluble calcium ions (Li et al., 2013). The bacterial community diversity index of cellar mud was negatively correlated with total acid and positively correlated with humus content and pH value (Huang et al., 2015). The production of flavor substances and precursors in the cellar mash is strongly associated with the predominant cellar flora and other microorganisms (Huimin, 2017; Li et al., 2016). For instance, the spatial arrangement of microbial abundance and diversity in the cellar corresponds to the distribution of volatile flavor compounds and their concentrations (Zhang, Zhang, et al., 2022). Moreover, *Clostridium* spp. and *Methanobacterium* spp. play a vital role in metabolizing essential aroma compounds and their precursors. The cellar mud displayed high diversity and species richness, indicating superior quality. Furthermore, the β‐diversity analysis revealed a significant correlation between the microbial community and the quality of the mud (Xiaolong, 2015).

The quality of cellar mud is mainly determined by the type and quantity of microorganisms in the cellar mud. Currently, most research on cellar sediment is focused on individual wineries like Wuliangye, with a lack of comprehensive studies on the ecological environment, brewing processes, and overall quality of cellar sediment within the production region. Large enterprises with greater resources started early, while small and medium‐sized enterprises lag behind. Small enterprises rely on the purchase of bacterial preparations from large liquor enterprises for production, resulting in the masking of geographical characteristics of strong‐flavored white wine, leading to an imbalanced development (Yifang, 1992).

This study takes the cellar mud of five enterprise wineries with different orientations and geographical environments in Yibin production area as the research object to analyze the quality and characteristics of cellar mud in Yibin production area. High‐throughput sequencing (next‐generation sequencing (NGS)) was used to identify microorganisms and their community structure. Headspace solid‐phase microextraction/gas chromatography–mass spectrometry hyphenation (HS‐SPME‐GC‐MS) was conducted for the study of volatile flavor substances and microbial correlations. The physicochemical indicators of the mud from the cellar illustrated that the quality of the mud varied across the production region of Yibin. The analysis of microbial community diversity and structure revealed significant variation in microbial community abundance within the Yibin production area, while the samples exhibited diverse volatile flavor substances. This paper examines the quality and exceptional attributes of liquor production in the Yibin area. It presents a comprehensive analysis of the different factors that influence the quality of cellar mud and their effect on the final product. This is beneficial for businesses seeking to enhance their mud and unique white wine styles within the appellation for the production of high‐quality liquor, to provide data reference and scientific basis.

## 2 MATERIALS AND METHODS

### 2.1 Sample collection

**FIGURE 1:**
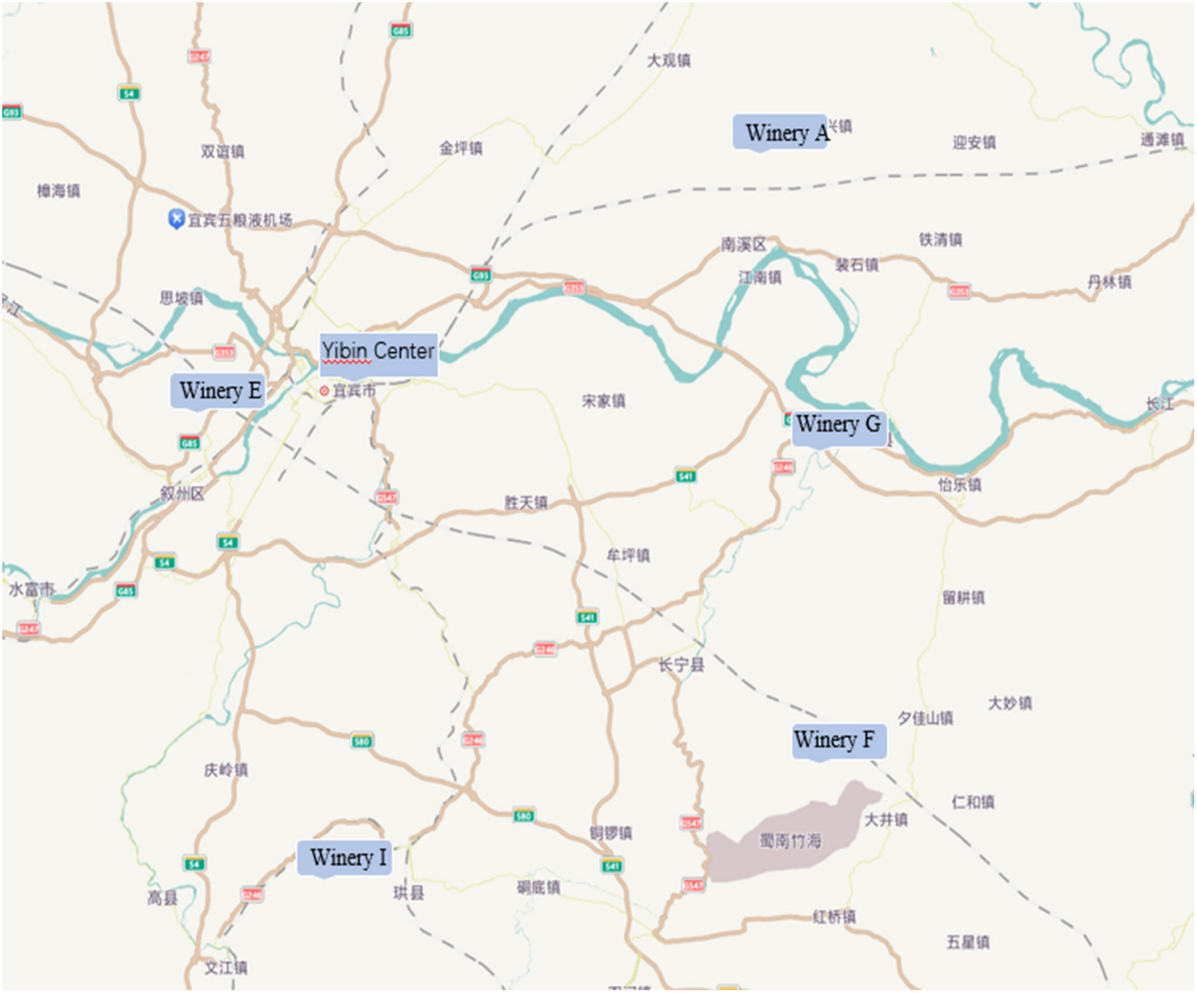
Geographical distribution map of winery.

The mud from three pits, aged approximately 15 years, was collected from five liquor producers in Yibin City, Sichuan Province, China, with establishment numbers A, E, F, G, and I (Figure 1). Three cellars of about 15 years of age were selected for each winery. Sampling points and methods concern the appropriate improvement (Wu et al., 2015; Zheng et al., 2013): After the annual cellar sweeping is completed, sterile stainless steel shovels are used to collect mud samples from each cellar. Mud samples are collected from a total of 13 points, including the upper, middle, and lower layers of each side of the cellar walls, as well as from the center of the cellar floor. Samples are taken at a depth of 5 cm from each layer, 8 cm from the ground flush part of the upper layer, and approximately 5 cm from the bottom of the cellar floor. The samples are stored in sterile polyethylene bags, sealed, and transported to the laboratory with dry ice. After arrival, they are placed in refrigerators at −20°C and −80°C for later use.

### 2.2 Determination of physicochemical indexes of the cellar mud

The moisture content of the cellar mud samples was determined using the method described by (Xie et al., 2018). Moisture content (%) was determined and reported as a percentage (Yifang, 1998). Tests for pH, ammonium nitrogen, effective phosphorus, and humus were conducted, according to the techniques outlined in Shen Yifang's Complete Book of White Wine Production Technology. The samples were divided into three portions where one fresh sample was used to determine moisture and ammonium nitrogen content, and one air‐dried sample was used to determine effective phosphorus and humus content. The cellar mud samples that were obtained were put into a white porcelain dish, with sample thickness not exceeding 2 cm; during the air‐drying period, it is necessary to turn and stir the cellar mud regularly. Additionally, larger pieces of cellar mud should be broken up and air‐dried in a cool area at natural room temperature without exposure to the sun. Once completely air‐dried, the mud should be preserved through a 60‐mesh sieve. Keep an additional sample for potential sample retesting or in the event of a dispute.

### 2.3 HS‐SPME‐GC‐MS analysis of volatile flavor substances in cellar mud

Weigh 1.0 g of the cellar mud sample in a headspace bottle (Zhang, Meng, et al., 2020) and add 2.00 g of sodium chloride (NaCl) and 5 mL of Ultrapure water (Xu et al., 2022). Then, add 10 μL of 2‐octanol standard and sonicate for 10 min. Insert the extraction needle and equilibrate at 50°C for 10 min. Next, extract and adsorb for 30 min, desorb at 230°C for 3 min, and analyze the sample using gas chromatography–mass spectrometry (GC–MS). GC conditions involved the use of helium as the carrier gas, with an inlet temperature set at 230°C. The sample was introduced into a non‐split injection with a J&W 122‐7062 column set at 250°C and dimensions specified as 60 m × 250 μm × 0.25 μm. The heating program followed an initial temperature of 40°C maintained for 5 min, with subsequent increase at 4°C/min to 100°C, then at 6°C/min to 230°C maintained for an additional 10 min. The MS conditions included an electron ionization (EI) source, with the ion source temperature set at 230°C.

The volatile flavor substances' mass spectra were compared to the standard spectrum library (NIST05a.L) using HS‐SPME‐GC‐MS for identification. The system's analysis software performed the flavor substances' analysis and data processing.

### 2.4 Analysis of microbial community structure and diversity in cellar mud

The entire genome from the samples was extracted using the methods described in the literature (Ohene‐Adjei et al., 2007; Xu et al., 2016); the bacterial isolate's 16S ribosomal RNA (rRNA) V3–V4 region underwent amplification via the use of primers 338F (5′‐ACTCCTACGGGGAGGCAGCAG‐3′) and 806R (5′‐GGACTACHVGGGTWTCTAAT‐3′). Amplifying the V3–V5 region of isolated Archaea sequences can be achieved by utilizing the Arch344F primer (5′‐ACGGGGGYGCAGCAGGCGCGA‐3′) and Arch915R primer (5′‐GTGCTCCCCCGCCAATTCCT‐3′), and all primers used were provided by Shanghai Meiji Biomedical Technology Co. Ltd. The primers consisted of 4 μL of 5 × FastPfu Buffer, 2 μL of 2.5 mM deoxynucleotide triphosphates (dNTPs), 0.8 μL of forward primer (5 μM), 0.8 μL of reverse primer (5 μM), 0.4 μL of FastPfu Polymerase, 0.2 μL of bovine serum albumin (BSA), and 10 ng of Template DNA. Finally, the solution was made up to 20 μL with double‐distilled water (ddH2O). These reagents were provided by Shanghai Meiji Biomedical Technology Co. Ltd. Polymerase chain reaction (PCR) conditions were: (a) 95°C pre‐denaturation for 3 min; (b) 95°C denaturation for 30 s; annealing for 30 s; and 72°C extension for 45 s, 25 cycles for bacteria and 35 cycles for archaea; and (c) 72°C extension for 10 min, 10°C until removed, and finally PCR amplification products were detected by 2% agarose gel electrophoresis, and sequencing of the PCR products was carried out in the IlluminaMiseq platform for bipartite sequencing by Shanghai Meiji Biomedical Technology Co. Ltd.

### 2.5 Statistical analysis

After overlapping the relationship with Illumina sequencing of PE reads data, quality control, de‐jointing, and filtering to obtain high‐quality sequences, OUT clustering of optimized sequences by Mothur software to obtain OUT abundance table, combined with statistical calculation of α‐diversity analysis, and the use of the R language, IBMSPSS26.0, and other software for data collation and plotting, analyze the resulting composition of cellar mud community and the structure of dominant flora. Sequencing analysis was also performed through the IlluminaMiSeq platform. The obtained operational taxonomic units (OTUs) were filtered to retain OTUs annotated to the bacterial and archaeal domains, and OTUs with an abundance of 1 and 2, as well as contaminating sequences (chloroplasts and mitochondria), were removed. Leveling was performed based on the minimum number of sample sequence pairs. The optimized sequences were grouped according to the default value of 97%, the dilution curves were plotted, and the shape of the curves was used to determine whether the current sequencing depth was sufficient to reflect the microbial diversity contained in the cellar soil samples of each winery.

## 3 RESULTS AND DISCUSSION

### 3.1 Determination of physicochemical properties of cellar mud

The physicochemical properties of cellar mud can be compared by water content, ammonium nitrogen, available phosphorus, humus, and pH. Appropriate moisture levels promote the growth, reproduction, and metabolism of microorganisms. However, excessive water can cause the cellar mud to become too thin. According to the figure, the moisture content of cellar mud samples from the five wineries varies. The average moisture content of the cellar mud is 36.80%. Wineries A and I have moisture contents below the average, measuring 6.60% and 16.93%, respectively. Meanwhile, Winery F has a moisture content higher than the average, measuring 5.87%. The difference between Wineries E and G is insignificant; both are higher than the average moisture content, measuring 5.79% and 11.14%, respectively. Comparing the moisture content of the cellars of all wineries to the standard moisture content for high‐quality cellars, Cellars E, F, and G fell within the expected range. In contrast, the remaining wineries were classified as normal cellars. The moisture content varied greatly among the different wineries, likely influenced by geographical location, environmental factors, and human factors. Overall, the winery cellars in the Yibin production area fall within the range of normal to high‐quality cellars. Distillery G is located in Jiang'an County, Yibin City, right next to the Yangtze River, while Distillery E is located in Xuzhou District, Yibin City, also next to the river and rich in water resources, which may be the reason for the high moisture content of their cellar soil. Distillery F is located in the Shunan Bamboo Sea, which has a humid climate with high rainfall. The dense vegetation, especially bamboo forests, covers the soil well, and the soil is mostly purple, which has good water retention properties and is able to effectively retain water and prevent water loss.

The pH of cellar mud microorganisms significantly affected their production and reproduction activities. The figure shows that the cellar soil pH of all five wineries ranged from 4.32 to 5.84, with an average pH of 5.00, indicating acidic conditions. The winery with the highest cellar soil pH was Winery I (pH = 5.84), while the lowest was Wineries F (pH = 4.44) and E (pH = 4.32). The winery with the highest cellar soil pH was Winery I (pH = 5.84), while the lowest was Wineries F (pH = 4.44) and E (pH = 4.32). The significance analysis results indicated significant variances (*p* < .05) in pH values among the distilleries. Distilleries A (pH = 5.38), G (pH = 5.04), and I exhibited pH values 7.60%, 0.8%, and 16.8% above the average pH, correspondingly. In contrast, Distilleries E and F had pH values 13.6% and 11.2% below the average pH, respectively. Moreover, Distillery I showed a 35.19% significant increase in pH values compared to Distillery E. According to Liu Yanmei and other researchers (Liu et al., 2019), pH levels between 4 and 6 are optimal for the growth of caproic acid bacteria. The analysis revealed that the acidity levels of environmental conditions in the cellars of the five wineries included in the study were suitable for caproic acid production and in line with the acidic environmental conditions required for the fermentation process of macrocystic acid and yeast. Distillery I is located in Gongxian County, Yibin City, where the soil parent material is complex and may contain a high level of alkaline substances, resulting in alkaline soil. In agricultural production, farmers may use some alkaline fertilizers or additives, which may change the pH value of the soil. The winery F is located in the Shunan Bamboo Sea, where the vegetation resources are very lush, the soil here is purple, and a large number of bamboo leaves fall to the ground and produce rich humus and minerals in the soil, which may have an impact on the soil's acidity, making it acidic, and thus lowering the pH value. The winery E is located in the Xuzhou District of Yibin City, where the soil type is dominated by acidic or strongly acidic yellow and yellow‐brown soils, which have a low pH value. Located in hilly and mountainous areas, the terrain is complex, and areas with large slopes are prone to soil erosion. Soil erosion leads to the loss of some alkaline substances in the soil, which lowers the soil's pH value.

Ammonium nitrogen serves as the primary source of nitrogen necessary for the production and proliferation of functional bacteria in cellar mud. The figure illustrates that the ammonium nitrogen concentration in the cellar mud varies from 63.14 mg/100 g to 92.50 mg/100 g, while the content in the cellar soil ranges from 63.14 mg/100 g to 92.50 mg/100 g with an average ammonium nitrogen content of 74.22 mg/100 g. The cellar mud of Winery A had the highest ammonium nitrogen content (92.50 ± 3.24 mg/100 g), while winery I had the lowest (63.14 ± 2.19 mg/100 g). The significance analysis results showed that the ammonium nitrogen content of the cellar samples from Distilleries A, G, and I was significantly higher and different than the average ammonium nitrogen content of the cellar soil samples. The results of significance analysis showed that the content of ammonium nitrogen in the sludge samples of distillery A, G and I was significantly higher than the average content of ammonium nitrogen by 24.63%, 5.59% and ‐14.93%, respectively. However, the differences in the cellar soil of Distillery F and Distillery G were not significant, as they were 1.25% and 2.7% higher than the average ammonium nitrogen content. Compared with other production areas, the ammonium nitrogen content of the cellar soil in the Yibin production area was higher than those in Anhui and Henan. The difference in cellar soil between the various production areas was significant, and the ammonium nitrogen content of the cellar soil in the Yibin production area was remarkably distinct. Distillery A is located in Yibin Changxing Township, an area of high agricultural activity, where farmers use nitrogen fertilizers to increase crop yields. These nitrogen fertilizers, when applied to the soil, may increase the effective nitrogen content of the soil.

Effective phosphorus is a critical component for microbial proliferation and soil reproduction. According to the diagram, the effective phosphorus levels of the five vineyards varied from 297.88 mg/100 g to 383.33 mg/100 g, with an average effective phosphorus content of 344.61 mg/100 g. The most substantial effective phosphorus concentration in the soil of the cellar was observed in Winery A (383.33 ± 20.29), while the minimum value was measured in Winery F (297.88 ± 14.31). The statistical analysis indicates that there is no significant difference in the effective phosphorus content between Wineries A, E, G, and I within the production area. These wineries showed a respective increase of 11.24%, 3.68%, 4.50%, and 5.26% above the average effective phosphorus content. Furthermore, the difference in effective phosphorus content between Wineries F and I was also not significant. Winery F exhibited a lower effective phosphorus content than the average by 13.56%. A comparison of previous studies revealed higher effective phosphorus content in cellar soil samples from Yibin distilleries compared to Anhui Luzhou‐flavored liquor cellar mud. Winery A, located in Changxing Township, Yibin City, is spread over a large number of hills and mountains that may be rich in phosphorus‐containing minerals, which are gradually released into the soil during weathering, erosion, and other natural processes, increasing the effective phosphorus content of the soil, and by nearby agricultural activities.

Humus and its decomposition products serve as the primary source of microbial nutrients. As depicted in the figure, the humus content in the cellar soil samples from the five wineries ranged between 8.00% and 15.95%, with an average value of 12.77%. The wineries with the highest humus content in their cellar soil were F (15.95% ± 0.31%) and E (15.40% ± 0.20%), whereas the lowest content was discovered in the Winery I (8.00% ± 0.21%). The significance analysis results revealed noteworthy variations in the cellar soil humus content among the various distilleries. Specifically, the humus content of Distilleries E and F was 20.52% and 20.60%, respectively, higher than the average content. Conversely, Distilleries A, G, and I exhibited 7.75%, 0.39%, and 37.35% lower humus content compared to the average. Compared with other production areas, Li Jiamin (Li et al., 1994) measured that the humus content in the cellar mud of the Tuo Bao distillery ranged from 9.7% to 14%. Compared with the humus content of the cellar mud of a Sichuan distillery of different vintages determined by Peng Kui (Peng et al., 2022), the humus content of Distilleries A, E, F, and G reached the standard of first‐class cellar mud; Zhang Huimin (Zhang, Wang, et al., 2020) measured the humus content of the cellar mud of a distillery in Sichuan of different years. He found that the humus content in the new cellar of a winery in Anhui was 18.61%, the humus content in the old cellar was 19.91%, and the humus content in the old cellar was higher than that in the new cellar, and the humus content in the five wineries in the Yibin production area was lower than that in Anhui (18.61%). Distillery F is located in the vicinity of the Shunan Bamboo Sea, which is located in the city of Yibin in the south of Sichuan Province. It is a natural scenic area dominated by bamboo forests, with vast bamboo forests and dense forest vegetation. This vegetation produces a large amount of organic matter through photosynthesis, which decomposes, transforms, and accumulates in the soil, forming a rich humus.

### 3.2 Cluster analysis of physical and chemical indexes of cellar mud

Based on the Euclidean distance algorithm, the physicochemical indexes of the cellar soil of five wineries were clustered and analyzed, and a heat map was drawn (Figure 3). The clustering results showed that moisture and humus were clustered together in one group, which was because the moisture and humus in the cellars of Wineries E, F, and G were not clustered together. The clustering results showed that moisture and humus were clustered into one group, which was due to the higher moisture and humus contents in the cellar soil of Wineries E, F, and G and the lower contents in Wineries A and I. The clustering results of pH and effective phosphorus were clustered into one group. The clustering of pH and effective phosphorus is due to the higher pH and effective phosphorus content in Wineries A and I and the lower pH and effective phosphorus content in Winery F. The pH and effective phosphorus contents were higher in Wineries A and I and lower in Winery F. The fact that Wineries A and I were clustered together and Wineries E, F, and G were clustered together indicates that the wineries in the production area can be categorized according to the physicochemical indicators. This indicates that the wineries in the production area can be classified into two groups based on physical and chemical indicators.

### 3.3 Analysis of microbial community structure and diversity of cellar mud in Yibin production area

High‐throughput sequencing technology was used to sequence the bacteria and archaea in the cellar mud, and the diversity of the bacterial and archaeal communities in the cellar mud was analyzed by ACE (Abundance‐based Coverage Estimator), Chao1, Shannon, Simpson, and Coverage indices, as well as the phylum‐level structure and genus‐level structure of the bacteria and archaea, which helped us to understand the characteristics of the composition of the microflora within the Yibin production area and to improve the understanding of the complex microbial structure of the wine cellar. The richness and diversity of microflora in the cellar mud of five wineries within the Yibin production area were analyzed by the Alpha Diversity Index, starting from the species of microbial communities in the samples, analyzing the species richness and homogeneity of the cellar mud samples of the factories, and investigating the diversity and coverage of the samples and whether they could represent the real micro‐ecological level in the samples. To further clarify the different genera in each winery, the sequenced microbial TOP20 dominant genera were subjected to difference significance analysis through statistical methods, and significant differences in microbial groups within the production area were screened out to provide further information for the production area. To further clarify the different microbial genera in each winery, the TOP20 dominant microbial genera after sequencing were analyzed by statistical methods, and the microbial groups with significant differences in the production area were screened out to provide a theoretical basis for further clarifying the influence of different microbial genera on the quality of cellar mud in the production area.

### 3.4 Rarefaction curve

After analyzing the high‐throughput sequencing results, the dilution profiles of the bacteria and archaea were plotted. Both the effective sequence and dilution curves can be used to characterize the diversity and richness of the microbial community of winery samples at different sequencing depths; if the curves are in an ascending phase, it indicates that the samples have not been sequenced to a sufficient depth and that more sequencing data are needed. When the trend of the curve is smoother, it means that the data can reflect all the species information in the samples. Bacteria were determined, and a total of 1,697,813 sequences were obtained from 15 cellar samples after optimizing the sequencing results, with an average length of 408.47 bp, of which 1,421,501 valid sequences were in the range of 401–440 bp, accounting for about 99. 61%, the valid sequences in the range of 410–420 bp accounted for about 83.72%, and the sequences in the range of 421–440 bp accounted for about 15.88%, which meets the sequencing requirements for bacterial samples. The optimized sequences were grouped according to the default value of 97%, and a total of 1369 OTUs were obtained. The sequences were extracted by a random sampling method, and a dilution curve was constructed with the number of OTUs the sequences could represent. Archaea were determined, and a total of 1,557,469 sequences were obtained from 15 cellar samples after optimized sequencing results, with an average length of 224.60 bp, of which 1,557,463 valid sequences were in the range of 201–240 bp, accounting for about 100.00%, valid sequences in the range of 200–220 bp (10,604) accounted for about 0.68%, and valid sequences in the range of 221–240 bp (1,546,859) accounted for about 99.32%, which met the sequencing requirements for bacterial samples of valid sequences (1,546,859) accounted for about 99.32%, which met the sequencing requirements for bacterial samples. The optimized sequences were grouped according to the default value of 97%, and a total of 44 OTUs were obtained. The sequences were extracted using a random sampling method, and the dilution curve was constructed with the number of OTUs that the sequences could represent. The results (as shown in Figure 5) show that the sequencing depth gradually tends to be parallel to the x‐axis after reaching 5000 sequences, indicating that the sampling volume is reasonable, the sample can truly reflect the composition of most of the microbial communities in the cellar mud, and the experimental results are real and reliable. Meanwhile, Figure 4 shows that the samples are rich in bacterial species and high in bacterial diversity, and the bacterial abundance of Distillery I is relatively low compared with those of other distilleries.

### 3.5 α–diversity analysis

Alpha diversity analysis was used to express both the species diversity and the richness of the microbial community in the samples. After conducting the statistical and bioinformatic analysis of the results of measuring the cellar mud samples in each winery, the sample diversity index table of bacterial microorganisms and archaeal microorganisms was obtained, and the coverage rate was over 99%, which indicated that the probability of measuring the cellar mud sequences in each winery in the production area was high, and the probability of not measuring was low, which indicated that the results of sequencing the samples could effectively reflect the real situation of each sample. The results showed that a total of 1,697,813 bacterial sequences were detected in the 15 cellar soil samples; the ACE index was the highest in Winery A, followed by E, F, and G, and the lowest in Winery I. The Shannon index, Chao1 index, and Simpson index were the highest in Winery A, indicating the highest bacterial community richness in the cellar soil. The Shannon index, Chao1 index, and ACE index were the highest, and the Simpson index was the lowest, which indicated that the diversity of bacterial community was the highest in Winery A, followed by the probability that the higher the index, the higher the degree of aging, which indicated that the degree of aging of cellar mud of Winery A might be higher than those of the other wineries. The coverage index of each sample was above 99.70%, which indicated that the probability of the sequence of cellar mud in each winery in the production area was high, and the probability of not being measured was low, which indicated that the sequencing results of the samples could effectively reflect the real situation of each sample, and the credibility of the data was high. A total of 1,557,469 archaeal sequences were detected in the 15 cellar samples, with the highest ACE and Chao1 indices in the sample from Winery E, indicating that it has the highest species diversity and richness and the highest Shannon and Simpson indices in Winery F, and the lowest in Winery I. This indicates that the species richness and diversity in each of the cellar samples in the production area are the highest. This suggests that the species richness and diversity of the archaeal communities in the cellar soils of the wineries in the production area are not identical (Tables 1 and 2).

**TABLE 1:** Diversity analysis of bacterial community structure in different wineries.

Sample ID	ACE	Chaol	Shannon	Simpson/%	Coverage/%
A	654.54 ± 14.62^a^	61.45 ± 13.43^a^	4.32 ± 0.02^a^	3.23 ± 0.10^b^	99.71 ± 0.02^b^
E	585.21 ± 22.81^ab^	591.14 ± 13.57^ab^	4.20 ± 0.15^ab^	3.50 ± 0.69^b^	99.72 ± 0.03^b^
F	522.52 ± 12.10^b^	519.63 ± 12.62^b^	3.82 ± 0.09^b^	5.78 ± 0.61^ab^	99.73 ± 0.03^b^
G	567.74 ± 53.64^ab^	560.44 ± 55.42^b^	4.14 ± 0.02^ab^	3.60 ± 0.47^b^	99.71 ± 0.04^b^
I	362.86 ± 87.99^c^	363.29 ± 80.86^c^	3.35 ± 0.46^c^	9.17 ± 6.38^a^	99.81 ± 0.08^a^

*Note*: These lowercase letters, different in the same line, represent a significant difference in the data in that line. Groups by different lowercase letters.

**TABLE 2:** Diversity analysis of archaeal community structure in different wineries.

Sample ID	ACE	Chaol	Shannon	Simpson	Coverage/%
A	41.31 ± 2.40^b^	40.03 ± 2.18^b^	1.78 ± 0.23^ab^	0.26 ± 0.09^ab^	99.99 ± 0.00^a^
E	74.01 ± 6.65^a^	70.61 ± 8.78^a^	1.57 ± 0.31^ab^	0.38 ± 0.14^ab^	99.99 ± 0.00^b^
F	55.09 ± 18.52^ab^	57.00 ± 18.77^ab^	1.88 ± 0.08^a^	0.22 ± 0.029^c^	99.99 ± 0.01^a^
G	62.35 ± 10.59^b^	50.95 ± 6.57^ab^	1.34 ± 0.08^bc^	0.36 ± 0.02^ab^	99.99 ± 0.00^b^
I	47.74 ± 12.00^b^	44.14 ± 11.23^ab^	1.04 ± 0.31^c^	0.45 ± 0.13^a^	99.99 ± 0.00^a^

*Note*: These lowercase letters, different in the same line, represent a significant difference in the data in that line. Groups by different lowercase letters.

### 3.6 Venn analysis

Venn diagrams are generally used to show the distribution of OUTs in the samples, which can clearly show the generalization and similarity of OUT composition in each sample. Based on the gate level analysis of common and specific bacterial gates in the cellar soil of different wineries in the production area Venn diagram a‐1, the results showed that 16 bacterial gates were detected in the production area, and nine bacterial gates were common to the cellar soil samples five wineries, which were Firmicutes, Bacteroidota, Synergistota, Actinobacteriota, Cloacimonadota, and unclassified_k_norank_d_Bacteria, the presence of two unique characteristic phyla, Thermotogota and Patescibacteria, only in winery G, suggests that winery G differs at the phylum level from the other four wineries of the appellation. Twelve bacterial phyla were found in the cellar mud of Wineries A, E, F, and G. Additionally, 13 phyla were observed in Wineries A, E, and G, and 14 phyla were found in Wineries A, G, and I. This demonstrates that the bacterial phyla present in the cellar mud of the wineries in the Yibin production area are relatively similar. The Venn diagram based on the analysis of common and special archaeal phyla from different wineries in the production area is presented in Figure 1. The study identified a total of four archaeal phyla in the production area, with four phyla being present in the cellar soil samples of all five wineries. The identified phyla were Euryarchaeota, Halobacterota, Thermoplasmatota, and unclassified_k__norank__d__Archaea, with no characteristic phyla indicating consistency among the cellar soil samples from the wineries in the Yibin production area.

### 3.7 Analysis of dominant microbial community structure in cellar mud

To further analyze the dominant bacterial phyla of microbial communities in the cellar mud of each winery in the production area, a horizontal bar chart of bacterial phyla was plotted based on the results of OUT statistics using the R language tool (Figure 7a). The figure displays that at the bacteriological level, the 15 samples contained six dominant phyla. The phyla from the highest to lowest were Firmicutes, Bacteroidota, Synergistota, Actinobacteriota, Cloacimonadota, and unclassified_k_norank_d_Bacteria. These results are comparable to those of the dominant phyla found in cellar sludge in some Yibin production area liquor breweries studied by previous researchers (Deng, 2015; You et al., 2012; Zhou et al., 2022).

Firmicutes accounted for more than 80% of the cellar soil in each winery, indicating that Firmicutes is the characteristic dominant bacterial phylum in the cellar soil of the wineries in the production area. The bacterial phylum Bacteroidota was the second most dominant in the cellar soil of Wineries A, E, and I, ranging from 1.88% to 9.22% in each winery. The largest percentage was found in Winery A. Furthermore, Bacteroidota was the most dominant bacterial phylum in the cellar soil of Wineries A, E, and I. Synergistota had the highest percentage (4.09%) of all bacterial populations found in the cellar soil of Winery G, while the lowest count was observed in Winery I (0.23%). Actinobacteriota exhibited a small distribution in each winery, ranging from 0.59% to 5.23% in the cellar soil of each winery. Among the wineries, the largest proportion of Actinobacteriota was found in Winery F, where it ranged from 0.59% to 5.23% in the cellar mud. Cloacimonadota was found to be more abundant in Vineyard E and, to a lesser extent, in the other districts. unclassified_k_norank_d_Bacteria was also found to be less abundant in the districts. Comparative analysis revealed that the differences in the levels of phyla within the production areas existed mainly in the two phyla Cloacimonadota and unclassified_k_norank_d_Bacteria, with large differences in the proportion of phyla in each winery, and Cloacimonadota accounted for the highest proportion of Cloacimonadota in Winery E. The compositional distribution of the dominant phyla was similar to the views of some scientists, and they were all dominated by thick‐walled phyla (Lei et al., 2023; Zhao et al., 2017).

Most of the microbial flora in the cellar sludge in the long‐term fermentation process gradually forms a complex and tends to stabilize the micro‐ecological environment. The micro‐ecological environment is complex and stabilized. According to the horizontal bar chart of bacterial genera (Figure 7b), the relative abundance of dominant bacterial genera in the cellar soil samples of each winery varied, and the distribution was different.

*Caproiciproducens* accounted for (11.60%–24.83%) of the cellar soil of each winery and was the largest genus in the cellar soil of Wineries E and F. The percentage of *Caproiciproducens* in the cellar soil of Wineries E and F was (11.60%–24.83%). *Syntrophaceticus* ranged from 2.56% to 16.41% across the distilleries and was the genus with the highest relative abundance in Distilleries A and G. The genus was the most abundant in Distilleries A and G. Norank__f__norank__o___norank__c__norank__p__Firmicutes were more distributed in the cellar soil of Winery I with a percentage of 24.48%, and the smallest in Winery F (0.56%). Hydrogenispora was also widely distributed among the distilleries (5.22%–8.18%), with the largest percentage (12.16%) in Winery A and the smallest in Winery G. Clostridium_sensu_stricto_12 was also widely distributed among the distilleries (1.14%–13.38%), especially in Distillery E with a relative abundance of 13.38%, which was the second most dominant genus in the distillery, and the least in Distillery I.

*Caproiciproducens* is the main microbial genus that produces caproic acid, which can produce other substances besides caproic acid through metabolisms, such as hydrogen, ethanol, and some organic acids, which is an important functional bacterium in strong‐flavored white wine (Gu et al., 2021), and is also the main synthesizer of ethyl caproate (Du et al., 2014), an important aromatic substance in white wine (Mingyang, 2007), and can also convert lactic acid into caproic acid, which makes a certain contribution to “increasing caproic acid and decreasing lactic acid” (Simpson, 1949). *Syntrophaceticus* is another important microbial genus in the cellar mud, a kind of anaerobic, that can metabolize the production of acetic acid of a class of bacteria; it has been proved that this type of bacteria with the consumption of H2 methanogenic bacteria co‐cultivated with acetic acid oxidation ability (Westerholm et al., 2010). In the cellar, it is mainly manifested in the mutual camping with the methanogenic archaea genus, indicating that there are methanogenic bacteria in the cellar sludge, further supporting the quality of cellar sludge in this production area tends to be old and mature(Cheng, 2019; Manzoor et al., 2016; Wang et al., 2020; Yan et al., 2020). Clostridium_sensu_stricto_12 is a carbon chain elongating microorganism directly related to the production of hexanoic acid(Liu et al., 2014). In addition, in the cellar soil of the five wineries in the production area, the more noteworthy bacterial genera also include *Lactobacillus*, whose main metabolite is lactic acid, one of the four important acids in white wine, which has the effect of reducing the pungent and astringent taste of wine in white wine and also increases the aftertaste of the wine, giving it an appropriate sweetness to improve the harmonization of the wine. Its main metabolite is lactic acid, one of the four important acids in liquor, which can reduce the pungent and astringent taste of liquor, increase the aftertaste, and give the liquor a suitable sweetness to improve the coordination of liquor; and ethyl lactate, which is closely related to lactic acid, is one of the important aroma substances in liquor, and to a certain extent, it plays a positive role in the quality and taste of liquor(Qian et al., 2020).

Archaea with an abundance ratio of less than 1% in all cellar mud samples were categorized as other, and a horizontal bar chart of archaeal phyla was created (Figure 7c). A total of four archaeal phyla were detected in the production area, and four phyla were common to the cellar soil samples of the five wineries, namely Euryarchaeota, Halobacterota, Thermoplasmatota, and unclassified_k__norank__d__Archaea, with no characteristic phyla, indicating that the species of archaeal phyla were consistent among the cellar soil samples of the wineries in the Yibin production area. To further explore the structure of the dominant archaeal microbial community in the cellar mud samples from each winery, the R language tool OTU statistics was used to draw a horizontal bar chart of archaeal genera (Figure 7d) and the relative abundance of less than 1% was attributed to Other. As shown in Figure 5d, the five dominant archaeal genera with relative abundance greater than 1% were *Methanobacterium*, *Methanoculleus*, *Methanosarcina*, *Methanobrevibacter*, and *Methanomassiliicoccus*, which were roughly the same as those of the cellar archaea studied by Liu Mauk (Mao et al., 2022; Liu et al., 2013; Zhang, Bai, & Zuo, 2022), and some of the differences may be due to the different detection techniques adopted, the different operations of personnel, and the different sampling methods and sampling locations selected. The percentage of *Methanobacterium* in each winery ranged from 40.27% to 82.69%, and it was the most dominant genus in Wineries A, E, F, and G and the second most dominant genus in Winery I. The percentage of *Methanoculleus* varied considerably in each winery (3.70%–50.16%), and it was higher in Wineries I and G, with 50.16% and 42.74%, respectively, and the lowest in Winery F (3.70%) (50.16% and 42.74%, respectively), and the lowest percentage (3.70%) in Winery F. *Methanosarcina* was the most abundant in Winery E (11.38%) and the least abundant in Winery G (2.66%). *Methanobrevibacter* was the most abundant in Winery A (20.31%) and the least abundant in Winery I (0.04%).

From the perspective of the overall archaeal genera, the dominant genera were mainly methanogens, among which *Methanobacterium* spp., *Methanoctococcum* spp., and *Pseudomona*s spp. were important indicator bacteria in the aged cellar mud (Chai et al., 2020; Lin et al., 2019; Liu, 2013; Stämmler et al., 2016; Tao et al., 2014; Vandeputte et al., 2017), which were detected in all the samples, and especially accounted for the largest share in Winery A. This indicated that the content of methanogens was richer in the wineries in the Yibin production area and that the muds of this cellar age were in the state of being aged or tending to be aged.

### 3.8 Analysis of volatile components of cellar mud

**FIGURE 2:**
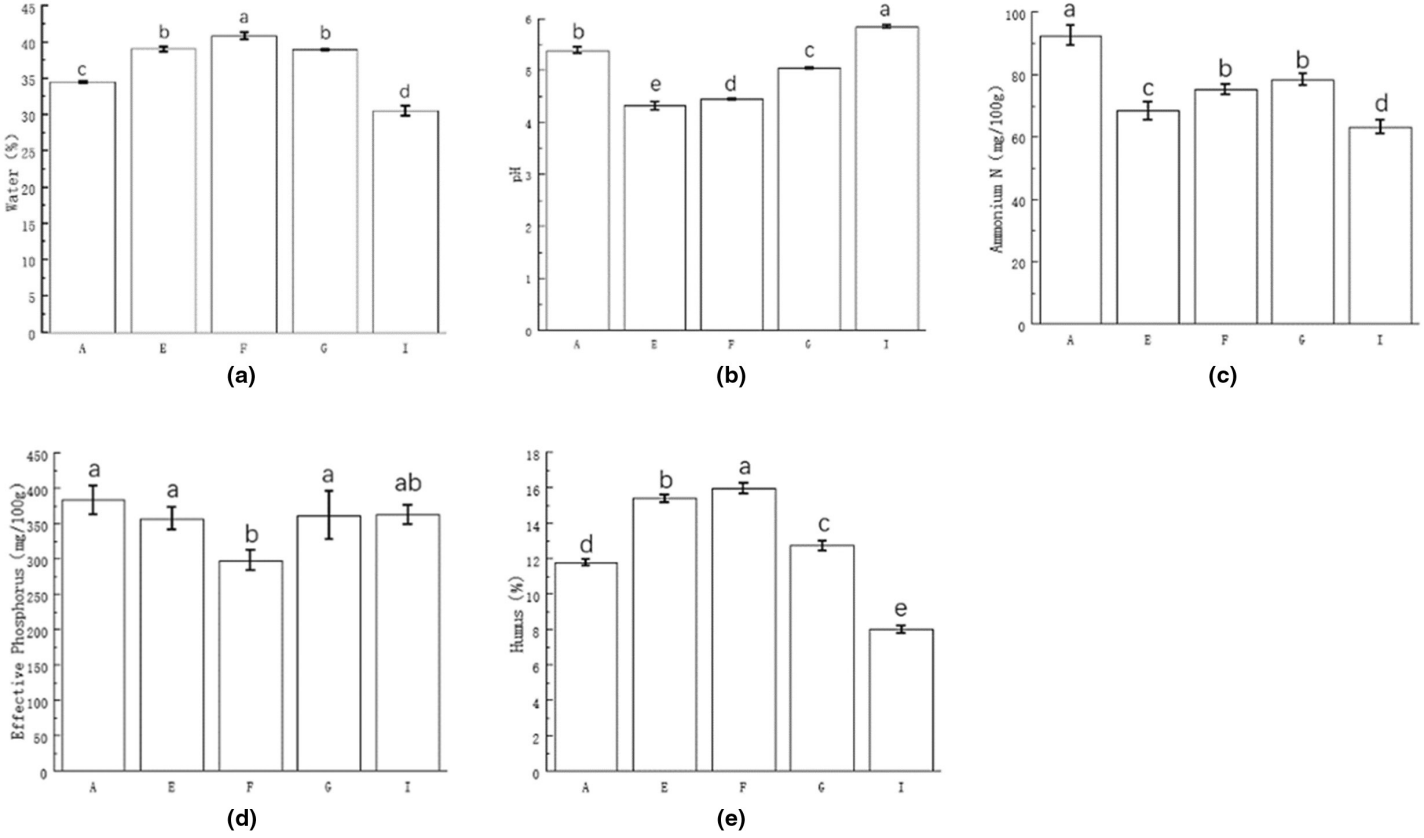
Physical and chemical results of cellar mud. (a) The moisture content of pit mud samples from various wine enterprises. (b) pH value of pit mud of each wine enterprise. (c) Ammonium nitrogen content in pit mud of various wine enterprises. (d) Available phosphorus content of each wine enterprise. (e) Humus content of each wine enterprise.

**FIGURE 3:**
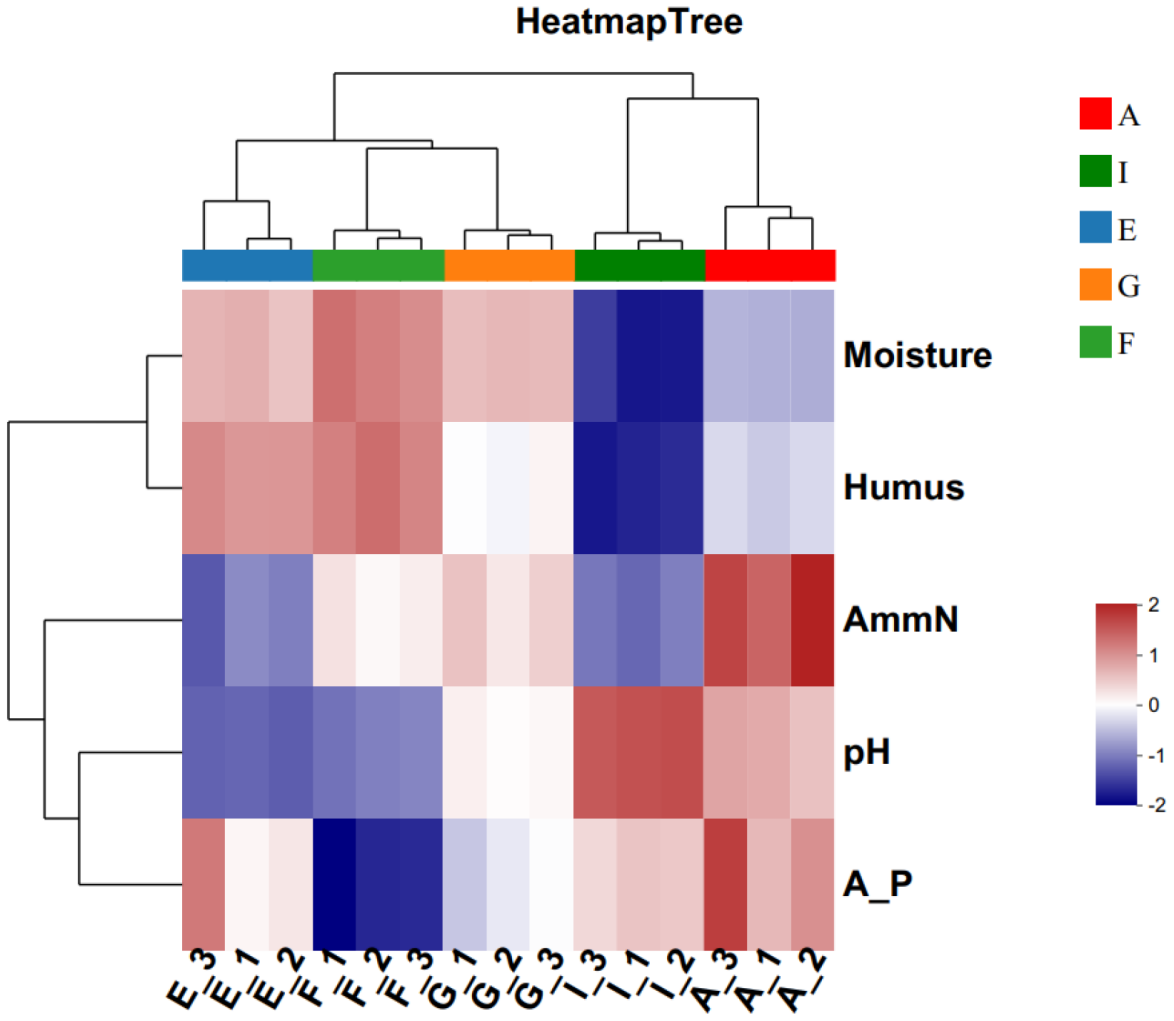
Cluster heat map of physicochemical indexes of pit mud in each winery.

**FIGURE 4:**
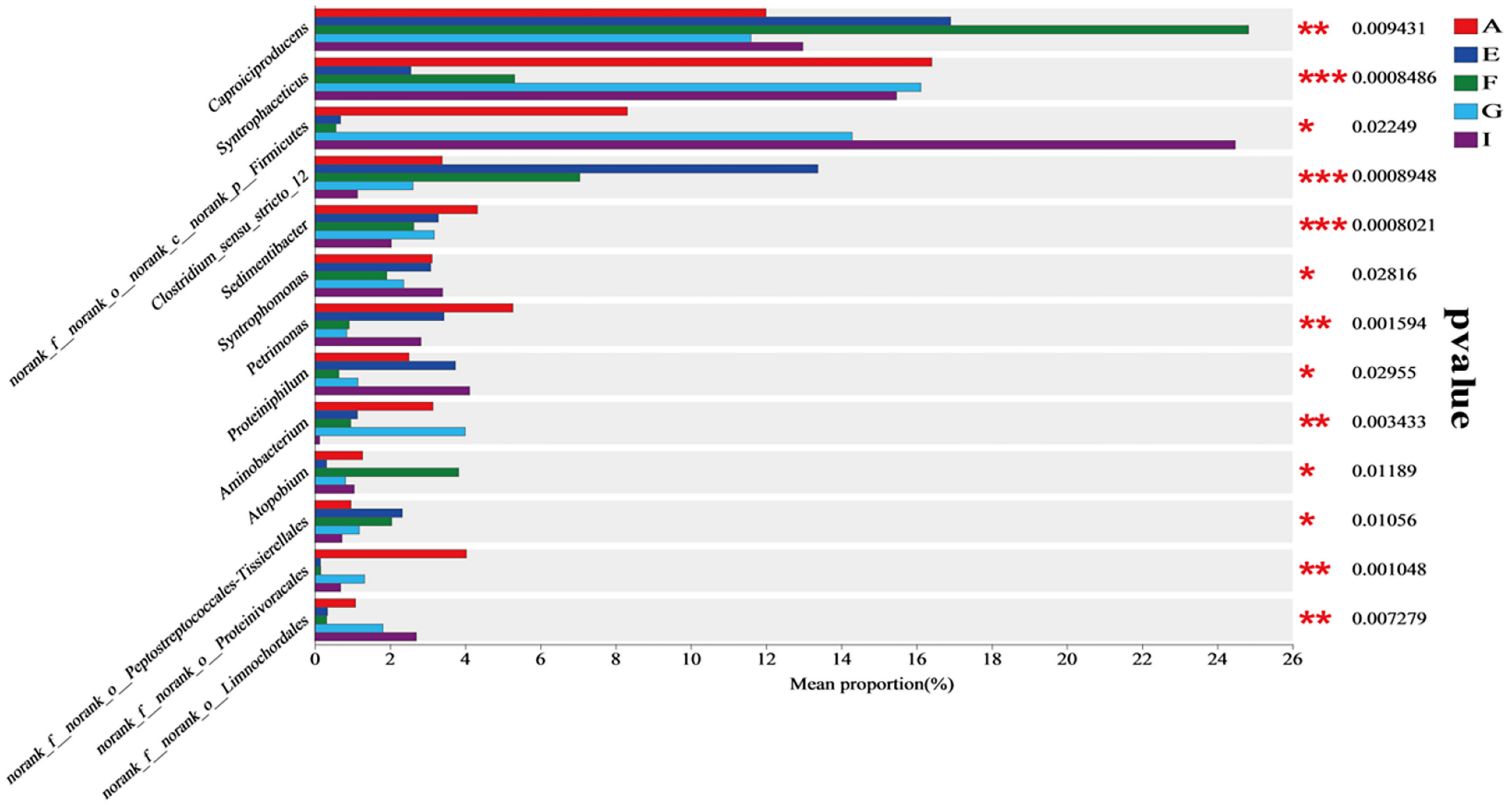
Significance analysis of differences in dominant bacterial groups.

**FIGURE 5:**
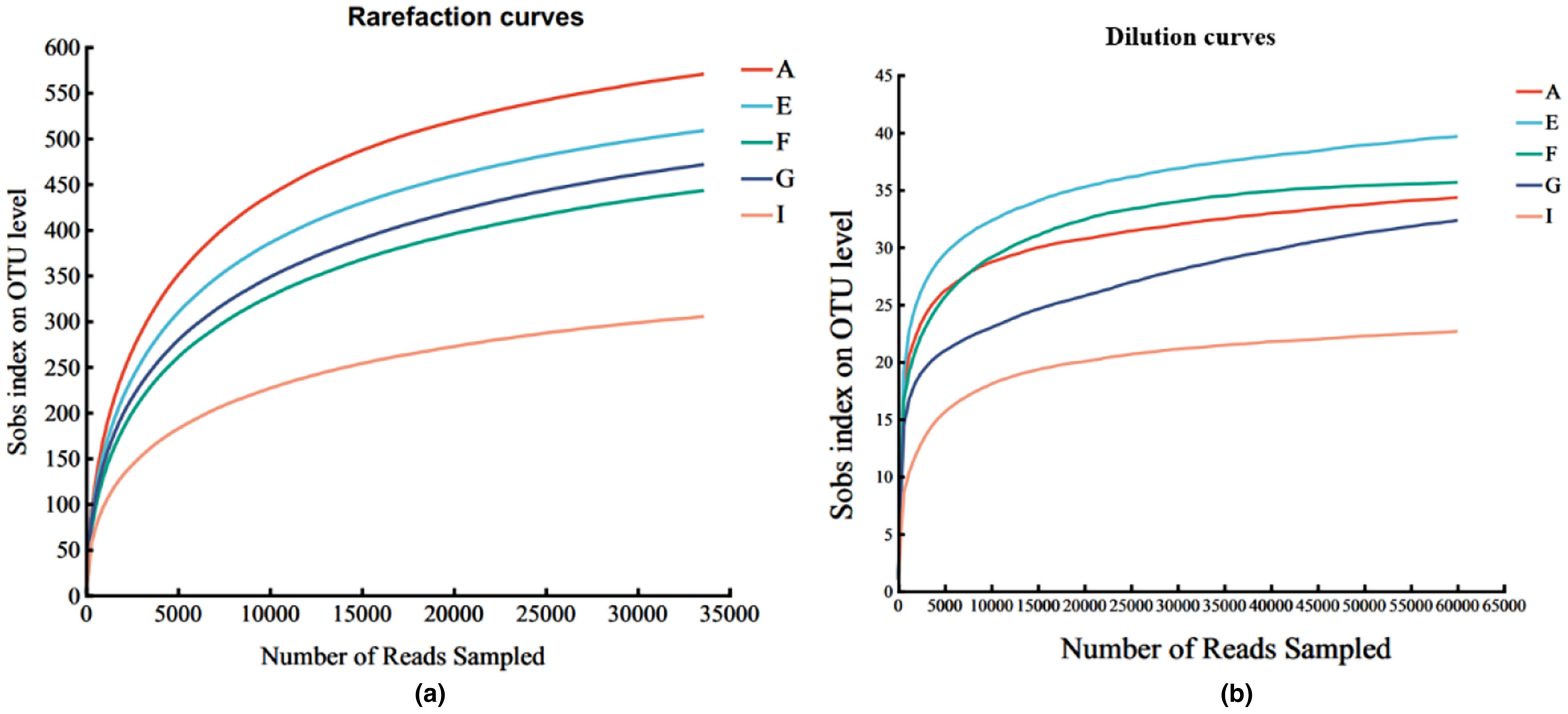
Evaluation of sequencing results (a) Rarefaction curve of bacteria. (b) Dilution curves of archaea.

**FIGURE 6:**
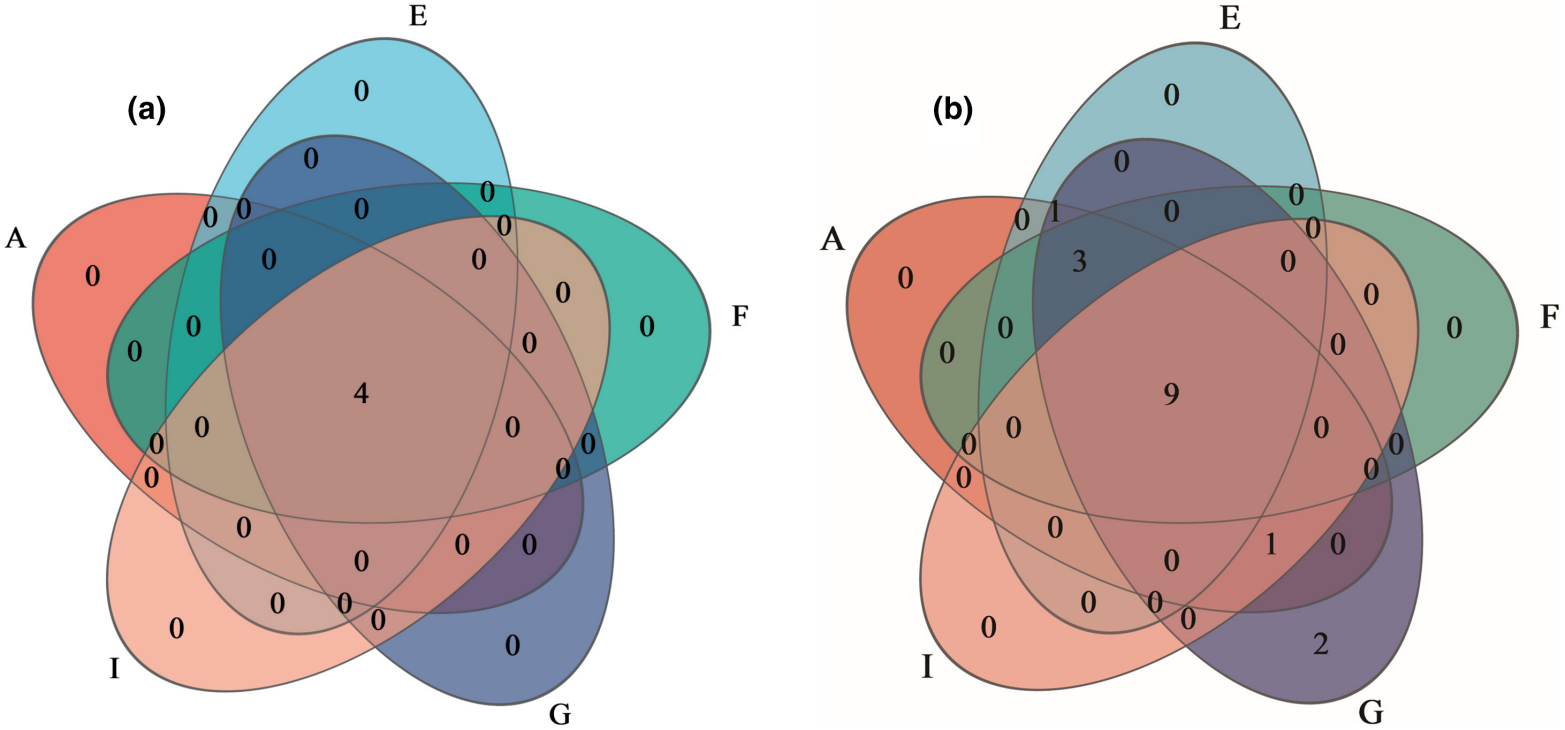
Venn analysis of five winery cellar species. (a) Bacteria; (b) Archaea.

**FIGURE 7:**
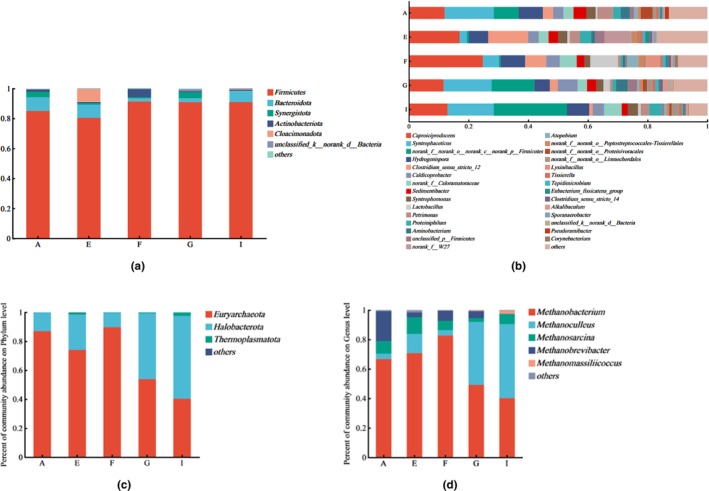
Analysis of microbial community structure of 15 samples of cellar mud. (a) Phylum level of dominant bacteria; (b) genus level of dominant bacteria; (c) Phylum level of dominant archaea; and (d) genus level of dominant archaea.

**FIGURE 8:**
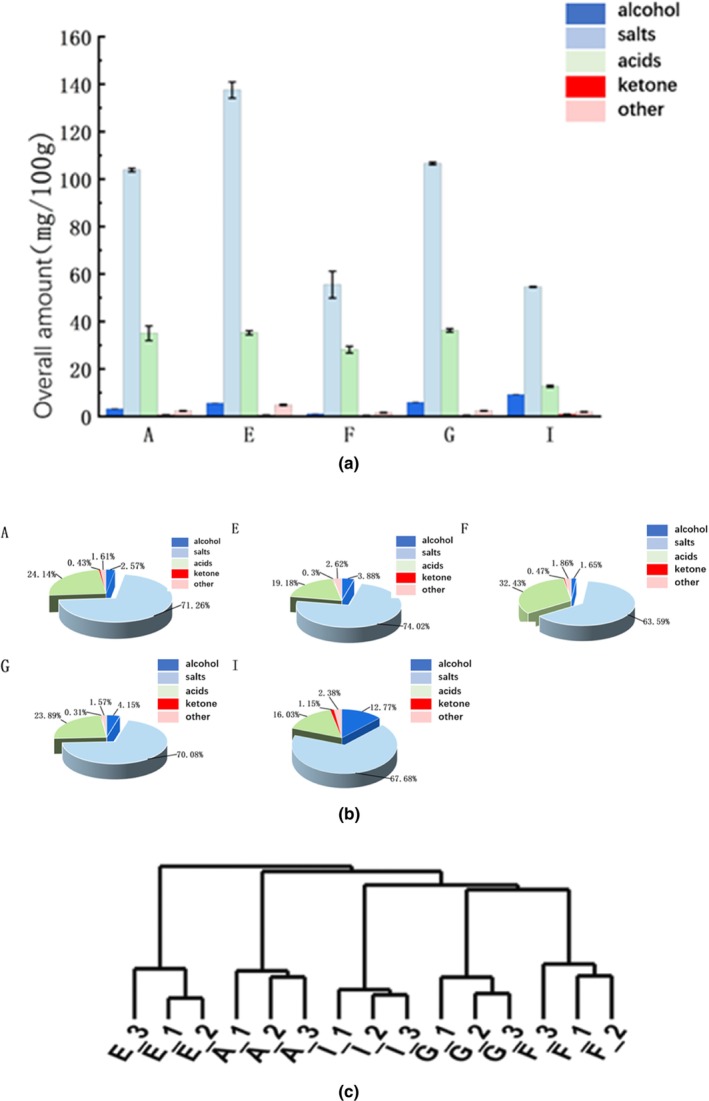
Comparison of volatile flavor compounds in pit mud. (a) Total volatile substances in cellar mud. (b) Percentage of main flavor substances. (c) Cluster analysis tree.

According to the determination results of the sample pit mud by HS‐SPME and GC‐MS, a total of 79 volatile flavor compounds were detected in the five cellar mash samples, of which 60 were found in Winery A, 41 in Winery E, 45 in Winery F, 49 in Winery G, and 50 in Winery I. The largest number of volatile flavor compounds was found in the cellar mash of Winery A. The volatile flavor compounds in the cellar mash were roughly composed of alcohols, esters, acids, aldehydes, and amides. The largest number of volatile flavor compounds was found in the cellar mash of Winery A. The volatile flavor compounds in the cellar mash were roughly composed of alcohols, esters, acids, aldehydes, ketones, and phenols. As shown in Figures 2, 3, 4, 5, 6, 7, 8, the cellar soil of the five wineries in the Yibin production area contained the highest content of esters, followed by acids and alcohols, which was consistent with the results of the previous study, and the detected volatile aroma compounds were similar to the aroma and flavor presenting substances in strongly flavored white wine (Tkacz et al., 2018; Zheng et al., 2019).

The ester content was the highest in all wineries. In terms of content, the highest ester content was found in the cellar soil of Winery E with 135.99 mg/100 g. The ester content of each winery accounted for more than 60% of the aroma compounds content of each winery, with ethyl caproate having the highest content. The highest acid content was found in Winery E (35.24 mg/100 g) and the lowest in Winery F (28.13 mg/100 g), indicating that more acid‐producing microorganisms may be present in Winery E. The higher acidity would cause a decrease in the pH in the cellar. Combined with the results of the physicochemical analysis, it was found (Figure 1b) that the cellar mud in Winery E had the lowest pH value, indicating that the test data were accurate and reliable. The content of alcohols was the third highest after esters and acids (Figure 7a), with the highest content in cellar I (10.12 mg/100 g), higher than the lowest content in cellar D (12.37 mg/100 g).

The esters in the cellar mud are the main aroma substances, mostly fruity and flora (Fan & Xu, 2011), which are mainly produced in two ways: slow esterification reaction and esterification enzymes in the aroma‐producing yeast in the cellar mud(Sun et al., 2013b), and their formation and transformation have a great influence on the flavor of the liquor(Shoubao et al., 2023; Zhang, Wu, et al., 2020). Ethyl caproate in the cellar soil was mainly produced by the reaction between caproic acid bacteria and ethanol, and the high content of ethyl caproate reflected that there were also more caproic acid‐producing microorganisms in the cellar soil, indicating that the dominant group of bacteria in the main body of wineries in the Yibin production area were caproic acid‐producing genera. The measurement results of acids showed that the content of acids in the cellar soil of each winery was second to that of esters, which is consistent with the characteristics of the proportion of acids in the cellar soil. As can be seen in Figure 6a, the acid content in the cellar soil of the other wineries in the production area, except winery F, was less than half the content of esters. Acids are the precursors for the generation of esters and are also the second largest volatile flavor substances in the cellar mud. Acids have an important influence on the main flavor of white wine and mainly play a role in softening and coordinating the body of the wine. Caproic acid is the precursor of the main flavor substances, which makes an important contribution to the flavor of the cellar mud and is also an important factor in the side reaction to the quality of the cellar mud (Zhou et al., 2022a); caproic acid is mainly derived from the coupling of methanogens and caproic acid bacteria, which ensures the oxidation of ethanol into the precursor acetic acid (Gao et al., 2021), and then promotes the generation of caproic acid by the methanogens (Zhang et al., 2019; Zhou, et al., 2022b; 2022a). The highest content of caproic acid was found in the cellar soil of each winery, indicating the presence of caproic acid bacteria and methanogens in the cellar soil of each winery. The winery with the highest percentage of alcoholic substances was Winery I, which accounted for 20.02% of the total amount of aroma substances within the winery, which was 6.6 times higher than the lowest percentage of Winery B. Alcoholic substances within each winery and the highest content of ethanol (Appendix A), the main aroma characteristics of alcoholic substances were floral, fruity, and alcoholic, n‐butanol and n‐amyl alcohol in the cellar soil had strong fruity and floral aroma, which mainly played the role of lining ester aroma in the body of wine, enriching the body of aroma, and some of the wineries, such as A and I, also had isoamyl alcohol with a very strong odor, which may be one of the important factors to differentiate the white wine aromas among the wineries (Fan & Xu, 2011).

Volatile flavor substances are mainly produced by the interaction of various microorganisms in the cellar, and the differences in the content of different types of volatile flavor substances in each brewery indicate that the microorganisms in the cellar differ in the type and metabolism of flavor substances, and they have different performances in different breweries, and the geographical location, the brewing process, and the operation of various units in the brewing process are not the same, which also affect the content of volatile flavor substances.

### 3.9 Cluster analysis of flavor substances

Based on the Euclidean distance algorithm, the volatile aroma compounds detected in the cellar mud of each winery were clustered, and the clustering tree was drawn using the R language. The results are shown in Figure 6c. The cellar mud samples of each winery could be clustered individually, indicating that there are obvious differences in the volatile aroma substances in the cellar mud of each winery in the production area. Among them, Distilleries G and F can be clustered together due to the low content of most of the volatile flavor compounds in the two distilleries. The results of the significance analysis of volatile flavor compounds in Table A1 further confirmed that the content of most volatile flavor compounds in the cellar soil of Distilleries G and F was close to each other and that there was a difference in volatile flavor compounds between the cellar soil of Distilleries G and F and that of the other distilleries. A total of 21 volatile aroma compounds, including n‐butanol, n‐hexanol, and ethyl hexanoate, were found to be statistically significantly different in the cellar soil samples of at least four wineries, suggesting that these compounds may be important factors contributing to the differences in cellar soil quality between different wineries and that they are the characteristic volatile aroma compounds in the cellar soil of different wineries within the production area.

## 4 CONCLUSION

In this study, the physicochemical properties of cellar mud samples from five different distilleries of Luzhou‐flavored liquor in the Yibin production area were investigated. HS‐SPME‐GC–MS was used to detect the microbial community structure and volatile components. The results showed that there were differences in the physicochemical indexes of cellar soil from each winery, and only the effective phosphorus content had small differences among wineries, but there were large differences in moisture content, ammonium nitrogen, humus, and pH value. Among them, the moisture content of the cellar soil in Wineries E, F, and G was higher, which was due to the characteristics of high‐quality cellar mud; the pH value of winery I was the highest, and the ammonium nitrogen content of Winery A was the highest, which was 24.63% higher than the average level; meanwhile, the effective phosphorus content of Winery A was also the highest, and the humus content of Winery F was the highest. The results of the microbial diversity survey and analysis showed that the dominant bacterial and archaeal community composition of each winery in the production area was relatively rich, with a high degree of similarity in community structure and greater commonality. However, there were also differences, with obvious regional differences in the cellar mud. A total of 13 genera of bacteria were present in the dominant genera of the TOP20, with the most in Wineries A and I, with four each, and one to two species in the cellar muds of the remaining wineries. Six dominant bacterial phyla, such as Firmicutes, Bacteroidota, and Synergistota, and 29 dominant bacterial genera, such as *Caproiciproducens*, *Syntrophaceticus*, and *Caldicoprobacter*, were detected, among which thick‐walled Bacteroidota was the dominant bacterial phyla, which was present in each winery, in the cellar sludge, and all of them accounted for more than 80.00%. Among the archaea, there were three different dominant archaeal genera, one each in Wineries A, I, and E. Among them, Euryarchaeota and Halobacterota were the dominant genera, with their relative abundance accounting for more than 97.52% in the respective samples. It is the presence of these differences that make up the characteristics of the white wine styles of the different wineries and distinguish them from other wineries. Among the 79 volatile aroma compounds detected in the cellar soil, esters were the most abundant, with ethyl caproate being the most abundant in all the cellar soil samples from the wineries. In terms of types, the cellar soil of Winery A contained the most volatile aroma compounds and that of Winery E the least. In terms of total amount, it was highest in Winery E (183.72 mg/100 g) and lowest in Winery I (78.27 mg/100 g). This indicates that there are differences in the types of microorganisms in the cellar and the aroma substances produced by their metabolism, which may be related to the differences in the microbial communities, and second, the geographical location, the brewing process, and the different operations of the units in the brewing process also affect the volatile aroma substances. For example, Wineries A and I are located in towns with more hilly terrain, more agricultural activities, lower pH, and higher effective phosphorus and ammonium nitrogen content in the cellar soil, and, therefore, more microbial species. Distillery A is located further north and has more volatile flavors in the cellar, while Distillery I is located the farthest south and has the lowest flavors in the cellar. The Wineries G and E are close to the river, so the moisture content of the cellar soil is high, which also affects the number of microorganisms and the content of flavor substances. Winery F is located in the Shunan Bamboo Sea in Yibin City, which has lush bamboo forest vegetation, a humid climate, sufficient moisture, and high soil humus. According to the data studied in this paper, it can be seen that the interaction of factors, such as climatic conditions, soil type, vegetation cover, topography, and agricultural activities, has a complex dynamic and interactive effect on the physicochemical indexes of the cellar soil, microbial population structure, and the content of flavor substances. The significance lies in that the style and characteristics of cellar mud in Yibin production area can be accurately and deeply understood through the research data. It is conducive to targeted research, not only for large wine enterprises, but also for small and medium‐sized wineries to better improve product quality and optimize their own processes. It can also find out the difference, so as to make up for its own shortcomings, and facilitate the adjustment and upgrading of the pit mud. And let the relevant practitioners have a more comprehensive understanding of the factors affecting the pit mud. Not just an extension from the old research direction, but an analysis in a more comprehensive and deeper way. Of course, only five representative cellar muds from wineries within the Yibin production area were selected as research samples in this paper. In the future, we will collect more samples from the Yibin production area and strengthen the experimental comparison of the main production areas of strong aromatic liquor outside Sichuan Province and the main production areas of Chengdu and Luzhou in Sichuan Province to provide data support for the comprehensive analysis of the unique quality of strong aromatic liquor in the Yibin production area and provide a scientific basis for the improvement of the quality of strong aromatic liquor.

## AUTHOR CONTRIBUTIONS

**Qiang Xu:** Writing – review and editing (equal). **Rangfang Chen:** Data curation (lead); methodology (equal); writing – review and editing (equal). **Minghong Bian:** Writing – review and editing (equal). **Hucheng Gong:** Writing – review and editing (equal). **Baolin Han:** Methodology (equal); supervision (equal); writing – review and editing (equal). **Shulin Tian:** Writing – review and editing (equal). **Yu Wang:** Writing – review and editing (equal). **Weitao Zhou:** Formal analysis (equal); writing – original draft (lead); writing – review and editing (equal).

## FUNDING INFORMATION

Sichuan Specialty Grain for Brewing Engineering and Technology Research Center‐Research on the Molecular Mechanism and Application of DHT7 in Regulating High Temperature Tolerance during the Rice Grouting Period (Grant number: E10527005), Chengdu Shuzhiyuan Liquor Industry Co., Ltd‐Research on Key Processes for Quality Improvement of Western Sichuan Strong‐Flavored Liquor (Grant number: HX2021034).

## CONFLICT OF INTEREST STATEMENT

We declare that we have no conflict of interest.

## ETHICS STATEMENTS

Consent to participate‐ not applicable.

## Data Availability

All data supporting the findings of this study are available within the paper and its Supplementary Information. R 4.2.2 software was used for data processing and visualization in this study. R software and its software packages are available from https://www.r‐project.org/.

## APPENDIX A

**TABLE A1:** The winery volatile flavor substance content (mg/100 g).

Volatile compound	Number	Name	CAS	A	E	F	G	I
Alcohol	1	Cyclobutanol	002919‐23‐5	0 ± 0^b^	0 ± 0^b^	0 ± 0^b^	0 ± 0^b^	4.82 ± 0.13^a^
	2	1‐Butanol	000071‐36‐3	0.11 ± 0.02^b^	0 ± 0^d^	0.06 ± 0.01^c^	0 ± 0^d^	0.21 ± 0.02^a^
	3	1‐Butanol, 3‐methyl‐	000123‐51‐3	0.05 ± 0.01^a^	0 ± 0^b^	0 ± 0^b^	0 ± 0^b^	0.05 ± 0.01^a^
	4	2‐Pentanol	006032‐29‐7	0 ± 0^b^	0 ± 0^b^	0 ± 0^b^	0 ± 0^b^	0.06 ± 0.01^a^
	5	1‐Pentanol	000071‐41‐0	0.09 ± 0.01^b^	0 ± 0^c^	0 ± 0^c^	0 ± 0^c^	0.24 ± 0.03^a^
	6	Isopropyl alcohol	000067‐63‐0	0.13 ± 0.03^a^	0 ± 0^b^	0 ± 0^b^	0 ± 0^b^	0 ± 0^b^
	7	1‐Pentanol, 4‐methyl‐	000626‐89‐1	0 ± 0^c^	0.27 ± 0.05^a^	0 ± 0^c^	0 ± 0^c^	0.08 ± 0.01^b^
	8	1‐Hexanol	000111‐27‐3	2.36 ± 0.08^c^	4.65 ± 0.07^a^	0.84 ± 0.05^d^	4.7 ± 0.23^a^	3.19 ± 0.08^b^
	9	2‐Heptanol, 6‐methyl‐	004730‐22‐7	0.08 ± 0.01^a^	0 ± 0^b^	0 ± 0^b^	0 ± 0^b^	0 ± 0^b^
	10	1‐Heptanol	000111‐70‐6	0.4 ± 0.02^c^	0.66 ± 0.07^b^	0.19 ± 0.02^d^	1.23 ± 0.04^a^	0.59 ± 0.06^b^
	11	2‐Dodecanol	010203‐28‐8	0.01 ± 0.01^a^	0 ± 0^b^	0 ± 0^b^	0 ± 0^b^	0 ± 0^b^
	12	2‐Heptanol, 5‐methyl‐	054630‐50‐1	0.01 ± 0.01^a^	0 ± 0^b^	0 ± 0^b^	0 ± 0^b^	0 ± 0^b^
	13	2‐Nonanol	000628‐99‐9	0 ± 0^b^	0 ± 0^b^	0 ± 0^b^	0 ± 0^b^	0.11 ± 0.02^a^
	14	1‐Octanol	000111‐87‐5	0.31 ± 0.03^c^	0.94 ± 0.12^a^	0.31 ± 0.06^c^	0.3 ± 0.03^c^	0.58 ± 0.06^b^
	15	2‐Pentanol, 3‐methyl‐	000565‐60‐6	0 ± 0^b^	0 ± 0^b^	0.03 ± 0.01^a^	0 ± 0^b^	0 ± 0^b^
	16	2,3‐Butanediol	000513‐85‐9	0 ± 0^b^	0.46 ± 0.02^a^	0 ± 0^b^	0 ± 0^b^	0 ± 0^b^
	17	1‐Nonanol	000143‐08‐8	0.04 ± 0.01^b^	0 ± 0c	0 ± 0^c^	0 ± 0^c^	0.11 ± 0.03^a^
	18	Phenylethyl alcohol	000060‐12‐8	0.13 ± 0.04^ab^	0.16 ± 0.03a	0 ± 0^d^	0.06 ± 0.01^c^	0.1 ± 0.01^bc^
Salts	19	Ethyl acetate	000141‐78‐6	0.49 ± 0.07^c^	0.64 ± 0.05^b^	0.48 ± 0.01^c^	0.57 ± 0.08^bc^	1.37 ± 0.06^a^
	20	Butanoic acid, ethyl ester	000105‐54‐4	0.83 ± 0.05^bc^	0.73 ± 0.02^c^	0.35 ± 0.04^d^	1.36 ± 0.09^a^	0.91 ± 0.07^b^
	21	Butanoic acid, 2‐methyl‐, ethyl ester	007452‐79‐1	0.04 ± 0.01^a^	0 ± 0^b^	0 ± 0^b^	0 ± 0^b^	0 ± 0^b^
	22	Formic acid, butyl ester	000592‐84‐7	0.11 ± 0.02^a^	0 ± 0^b^	0 ± 0^b^	0.13 ± 0.02^a^	0 ± 0^b^
	23	Acetic acid, pentyl ester	000628‐63‐7	0.03 ± 0.01^a^	0 ± 0^b^	0 ± 0^b^	0 ± 0^b^	0 ± 0^b^
	24	Butanoic acid, butyl ester	000109‐21‐7	0 ± 0^c^	0 ± 0^c^	0.06 ± 0.01^b^	0.05 ± 0.02^b^	0.12 ± 0.01^a^
	25	Hexanoic acid, methyl ester	000106‐70‐7	0.05 ± 0.01^a^	0 ± 0^b^	0 ± 0^b^	0 ± 0^b^	0 ± 0^b^
	26	Pentanoic acid, 4‐methyl‐, ethyl ester	025415‐67‐2	0.09 ± 0.01^a^	0 ± 0^b^	0 ± 0^b^	0 ± 0^b^	0 ± 0^b^
	27	1‐Butanol, 3‐methyl‐, formate	000110‐45‐2	0.06 ± 0.02^a^	0 ± 0^b^	0 ± 0^b^	0 ± 0^b^	0 ± 0^b^
	28	Pentanoic acid, propyl ester	000141‐06‐0	0.16 ± 0.02^a^	0 ± 0^b^	0 ± 0^b^	0 ± 0^b^	0 ± 0^b^
	29	Hexanoic acid, ethyl ester	000123‐66‐0	63.73 ± 0.46^a^	52.06 ± 3.12^b^	25.02 ± 0.44^d^	61.31 ± 0.75^a^	28.82 ± 0.12^c^
	30	Formic acid, pentyl ester	000638‐49‐3	0.12 ± 0.02^a^	0.12 ± 0.02^a^	0 ± 0^b^	0 ± 0^b^	0 ± 0^b^
	31	Propanoic acid, 2‐methyl‐, 3‐methylbutyl ester	002050‐01‐3	0 ± 0^b^	0 ± 0^b^	0.03 ± 0.01^a^	0 ± 0^b^	0 ± 0^b^
	32	Butanoic acid, 3‐methylbutyl ester	000106‐27‐4	0 ± 0^b^	0 ± 0^b^	0 ± 0^b^	0 ± 0^b^	0.03 ± 0.01^a^
	33	Acetic acid, hexyl ester	000142‐92‐7	0.25 ± 0.01^bc^	0.26 ± 0.04^bc^	0.14 ± 0.01^c^	1.31 ± 0.12^a^	0.38 ± 0.09^b^
	34	2‐Heptanone	000110‐43‐0	0.68 ± 0.03^b^	0 ± 0^d^	0.41 ± 0.01^c^	0.47 ± 0.04^c^	0.93 ± 0.09^a^
Salts	35	Hexanoic acid, 2‐methylpropyl ester	000105‐79‐3	0 ± 0^c^	0.32 ± 0.03^b^	0 ± 0^c^	0.7 ± 0.08^a^	0.29 ± 0.05^b^
	36	Heptanoic acid, ethyl ester	000106‐30‐9	6.27 ± 0.16^b^	7.75 ± 0.29^a^	3.87 ± 0.06^c^	6.37 ± 0.12^b^	2.45 ± 0.22^d^
	37	Propanoic acid, 2‐hydroxy‐, ethyl ester	000097‐64‐3	0.12 ± 0.02^c^	0.46 ± 0.02^a^	0.22 ± 0.02^b^	0.24 ± 0.09^b^	0.12 ± 0.02^c^
	38	Formic acid, hexyl ester	000629‐33‐4	2.43 ± 0.06^b^	2.68 ± 0.03^a^	0.84 ± 0.05^d^	1.77 ± 0.12^c^	0 ± 0^e^
	39	Pentanoic acid, 3‐methylbutyl ester	002050‐09‐1	0.04 ± 0.01^a^	0 ± 0^b^	0.03 ± 0.01^a^	0.04 ± 0.01^a^	0 ± 0^b^
	40	Acetic acid, heptyl ester	000112‐06‐1	0.04 ± 0.01^a^	0 ± 0^b^	0 ± 0^b^	0.03 ± 0^a^	0.03 ± 0.01^a^
	41	Hexanoic acid, 2‐methylbutyl ester	002601‐13‐0	0 ± 0^b^	0 ± 0^b^	0 ± 0^b^	0.46 ± 0.01^a^	0 ± 0^b^
	42	2H‐Pyran‐2‐one, tetrahydro‐6‐propyl‐	000698‐76‐0	0.19 ± 0.01^b^	0.25 ± 0.03^a^	0.25 ± 0.03^a^	0 ± 0^c^	0 ± 0^c^
	43	Hexanoic acid, butyl ester	000626‐82‐4	4.08 ± 0.05^b^	5.62 ± 0.19^a^	2.46 ± 0.12^c^	2.11 ± 0.04^d^	2.29 ± 0.15^cd^
	44	Octanoic acid, ethyl ester	000106‐32‐1	9.52 ± 0.02^c^	27.25 ± 0.11^a^	7.03 ± 0.08^d^	10.14 ± 0.02^b^	4.69 ± 0.07^e^
	45	Formic acid, heptyl ester	000112‐23‐2	0.4 ± 0.02^a^	0.22 ± 0.04^b^	0.19 ± 0.01^b^	0 ± 0^c^	0 ± 0^c^
	46	Isopentyl hexanoate	002198‐61‐0	0.81 ± 0.13^c^	1.62 ± 0.1^a^	1.09 ± 0.1^b^	0.83 ± 0.01^c^	0.63 ± 0.07^d^
	47	Undecanoic acid, ethyl ester	000627‐90‐7	0 ± 0^d^	0.2 ± 0.03^a^	0.06 ± 0.02^bc^	0.07 ± 0.02^b^	0.04 ± 0.01^c^
	48	Hexanoic acid, pentyl ester	000540‐07‐8	1.46 ± 0.04^bc^	3.39 ± 0.09^a^	1.14 ± 0.4^cd^	1.52 ± 0.06^b^	1.04 ± 0.09^d^
	49	Propyl octanoate	000624‐13‐5	0.23 ± 0.04^a^	0 ± 0^c^	0.12 ± 0.03^b^	0.19 ± 0.02^a^	0.08 ± 0.02^b^
Salts	50	Nonanoic acid, ethyl ester	000123‐29‐5	0.53 ± 0.02^b^	1.3 ± 0.02^a^	0.42 ± 0.2^b^	0.47 ± 0.05^b^	0.25 ± 0.05^c^
	51	Heptanoic acid, 3‐methylbutyl ester	000109‐25‐1	0.06 ± 0.01^a^	0 ± 0^b^	0 ± 0^b^	0.06 ± 0.01^a^	0 ± 0^b^
	52	Hexanoic acid, hexyl ester	006378‐65‐0	9.27 ± 0.16^bc^	24.92 ± 0.18^a^	9.2 ± 5^bc^	12.94 ± 0.13^b^	8.54 ± 0.2^c^
	53	Decanoic acid, ethyl ester	000110‐38‐3	0.41 ± 0.05^c^	1.91 ± 0.02^a^	0.5 ± 0.2^c^	0.76 ± 0.1^b^	0.47 ± 0.07^c^
	54	Octanoic acid, 3‐methylbutyl ester	002035‐99‐6	0 ± 0^b^	0 ± 0^b^	0 ± 0^b^	0.08 ± 0.01^a^	0 ± 0^b^
	55	Benzeneacetic acid, ethyl ester	000101‐97‐3	0.34 ± 0.02^b^	0.24 ± 0.04^a^	0.15 ± 0.09^d^	0.45 ± 0.04^c^	0.09 ± 0.01^d^
	56	Octanoic acid, hexyl ester	001117‐55‐1	0 ± 0^d^	3.92 ± 0.1^a^	0.91 ± 0.07^c^	1.57 ± 0.16^b^	0 ± 0^d^
	57	Furfuryl hexanoate	039252‐02‐3	0.1 ± 0.01^b^	0.11 ± 0.02^ab^	0.16 ± 0.06^a^	0.04 ± 0.02^c^	0.04 ± 0.01^c^
	58	Benzenepropanoic acid, ethyl ester	002021‐28‐5	0.39 ± 0.02^a^	0.13 ± 0.06^b^	0 ± 0^c^	0.12 ± 0.02^b^	0.05 ± 0^c^
	59	Octanoic acid, octyl ester	002306‐88‐9	0 ± 0^a^	0 ± 0^b^	0 ± 0^b^	0.06 ± 0.01^b^	0 ± 0^b^
	60	Hexadecanoic acid, ethyl ester	000628‐97‐7	0 ± 0^b^	0 ± 0^b^	0.03 ± 0.01^a^	0 ± 0^b^	0 ± 0^b^
Acids	61	Acetic acid	000064‐19‐7	0.26 ± 0.01^cd^	0.34 ± 0.05^bc^	0.75 ± 0.05^a^	0.39 ± 0.07^b^	0.25 ± 0.01^d^
	62	Propanoic acid	000079‐09‐4	0.06 ± 0.02^a^	0.03 ± 0.01^c^	0.05 ± 0.02^c^	0.05 ± 0^ab^	0.03 ± 0.01^ab^
	63	Butanoic acid	000107‐92‐6	1.46 ± 0.05^ab^	0.91 ± 0.01^b^	1.81 ± 0.72^a^	1.81 ± 0.16^a^	1.07 ± 0.16^b^
	64	Pentanoic acid, 3‐methyl‐	000105‐43‐1	0 ± 0	0.52 ± 0.72	0 ± 0	0 ± 0	0 ± 0
	65	Butanoic acid, 3‐methyl‐	000503‐74‐2	0.17 ± 0.06^c^	0 ± 0^c^	0 ± 0^c^	0.69 ± 0.08^a^	0.27 ± 0.04^b^
Acids	66	Pentanoic acid	000109‐52‐4	0.66 ± 0.08^ab^	0.66 ± 0.11^ab^	0.89 ± 0.52^a^	0.82 ± 0.07^a^	0.31 ± 0.08^b^
	67	Pentanoic acid, 4‐methyl‐	000646‐07‐1	0 ± 0^c^	0 ± 0^c^	0.05 ± 0.02^a^	0 ± 0^c^	0.02 ± 0.01^b^
	68	Hexanoic acid	000142‐62‐1	24.83 ± 2.74^b^	23.78 ± 0.54^b^	20.72 ± 0.1^c^	27.26 ± 0.49^d^	9.13 ± 0.17^a^
	69	Heptanoic acid	000111‐14‐8	3.66 ± 0.08^b^	4.53 ± 0.12^a^	2.09 ± 0.06^d^	2.8 ± 0.15^c^	0.72 ± 0.12^e^
	70	Octanoic acid	000124‐07‐2	3.61 ± 0.1^b^	4.48 ± 0.15^a^	1.67 ± 0.05^d^	2.33 ± 0.1^c^	0.86 ± 0.11^e^
	71	Nonanoic acid	000112‐05‐0	0.19 ± 0.1^a^	0 ± 0^c^	0.1 ± 0.05^ab^	0.07 ± 0.02^bc^	0 ± 0^c^
	72	n‐Decanoic acid	000334‐48‐5	0.1 ± 0.02^a^	0 ± 0^c^	0 ± 0^c^	0 ± 0^c^	0.04 ± 0.01^b^
Ketone	73	2‐Octanone	000111‐13‐7	0.62 ± 0.06^b^	0.55 ± 0.01^c^	0.4 ± 0.03^d^	0.45 ± 0.01^d^	0.88 ± 0.05^a^
	74	2‐Nonanone	000821‐55‐6	0 ± 0^b^	0 ± 0^b^	0 ± 0^b^	0.02 ± 0.01^a^	0.03 ± 0.02^a^
	75	Phenol	000108‐95‐2	0.23 ± 0.02^ab^	0.25 ± 0.01^a^	0.08 ± 0.02^b^	0.18 ± 0.19^ab^	0.1 ± 0.01^ab^
	76	Phenol, 4‐methyl‐	000106‐44‐5	1.23 ± 0.01^b^	1.76 ± 0.21^a^	0.75 ± 0.05^c^	0.74 ± 0.07^c^	0.73 ± 0.02^c^
Others	77	Phenol, 2,4‐bis(1,1 dimethylethyl)‐	000096‐76‐4	0.73 ± 0.1^c^	2.69 ± 0.33^a^	0.73 ± 0.08^c^	1.43 ± 0.08^b^	1 ± 0.07^c^
	78	Hexanal	000066‐25‐1	0.07 ± 0.01^a^	0 ± 0^b^	0 ± 0^b^	0 ± 0^b^	0 ± 0^b^
	79	Benzaldehyde	000100‐52‐7	0.07 ± 0.01^b^	0.11 ± 0.04^a^	0.05 ± 0.01^bc^	0.02 ± 0.01^c^	0.05 ± 0.01^bc^

*Note*: These lowercase letters, different in the same line, represent a significant difference in the data in that line. Groups by different lowercase letters.
